# “You are not alone”: Family-based HIV risk and protective factors for Hispanic/Latino men who have sex with men in San Juan, PR

**DOI:** 10.1371/journal.pone.0268742

**Published:** 2022-06-16

**Authors:** Moctezuma Garcia

**Affiliations:** 1 School of Social Work, San José State University, San José, CA, United States of America; 2 Research Education Institute for Diverse Scholars (REIDS), Center for Interdisciplinary Research on AIDS (CIRA), Yale University, New Haven, CT, United States of America; UNITED STATES

## Abstract

Hispanic/Latino men who have sex with men (MSM) have the second largest HIV infection rate in the United States and Puerto Rico (PR) has ranked number five with the greatest number of Hispanics living with HIV. This study aims to understand how family affects HIV risk and protective factors for young adults. PR MSM ages 21 through 30 in San Juan, PR completed semi-structured interviews exploring the influence interpersonal family relationships have on HIV risk and protective factors. PR MSM (N = 15) completed a semi-structured in-depth individual interview. NVivo was used for administering a thematic analysis based on the transcripts in the original language of the interview, 14 in Spanish and one in English. The following five general themes emerged from the data analysis: 1) Immediate versus Extended Family; 2) The Matriarch; 3) Fractured Paternal Relationships; 4) Siblings Influence; and 5) *Fictive Kin*–Creating My Own Family. Findings suggest that the immediate family play an integral role in enhancing HIV protective factors for PR MSM as young adults. This study highlights the importance for developing family-based interventions that reinforce cultural beliefs and values through a strengths-based approach towards enhancing HIV protective behaviors for PR MSM.

## Introduction

HIV continues to persist despite significant medical advances in treatment (antiretroviral medications) and prevention (e.g., pre-exposure prophylaxis), which is primarily concentrated among Black and Hispanic/Latino (hereafter Hispanic is used as a gender neutral term to reference people that identify with the diverse Spanish-speaking diaspora) men who have sex with men [MSM; 1]. The United States (US) government has proposed an aggressive strategy for Ending the HIV Epidemic focused on the highest burdened counties [1, 2]. Puerto Rico (PR) has the fifth largest number of Hispanic People Living With HIV (PLWH) in the US [3]. San Juan, PR has been identified as an HIV burdened area; 81.9% of PLWH are males and 57.4% reported MSM as the primary mode of HIV transmission [4]. HIV related research among sexual minorities of color has focused on deficiencies and limited research has taken a culturally informed approach regarding identity development and interpersonal family relationships [5–7]. Family support for Hispanic MSM has been identified as a resilient factor for reducing the risk of HIV exposure [8, 9] as well as medical adherence for PLWH [9, 10]. However, Hispanic MSM have also reported that their family is an important source of negative mental health outcomes (e.g., stress, anxiety, depression) due to concerns of being stigmatized based on their sexuality, gender, and HIV status [11–13]. People that encounter stigmas associated with race, sexuality, and HIV are more likely to endure greater health disparities [7, 14–18].

Sexual minorities expressed that a fear of being stigmatized by family or friends prevents them from disclosing their sexuality and/or HIV status, which increases one’s risk behavior and exposure to sexually transmitted infections [9, 18–20]. Adolescents that reported being rejected by their parents due to one’s sexual orientation were more likely to have greater levels of substance use, depression, suicidal ideation, suicide attempts, and sexual risk behaviors [21]. Sexual minorities that reported lower levels of parent conflict were less likely to have high-risk behaviors [22]. The research [6, 22, 23] demonstrates a strong association on the parent-child relationship with sexual minorities, but there is a significant gap in the literature focusing on Hispanic MSM ages 21–30.

Overall, the limited research available on family places an emphasis on family as a unit and is primarily based on the heterosexual Mexican community [9]. Several researchers have explored the implications of family for HIV related factors among sexual minorities, but it has been primarily on youth (ages 15–24), and Hispanics are usually a subsample of the study. Approximately 52% of Hispanic MSM were diagnosed with HIV between the ages of 20–29 [24], which warrants a qualitative approach to gain an understanding of HIV-related factors to develop culturally informed interventions for young adult Hispanic MSM. It is important to take into consideration that a defined age cohort for young adulthood varies due to cognitive as well as psychosocial milestones influenced by age and culture [25]. Therefore, the age range for young adults in this study is from 21 through 30 to explore the significance of family, prescribed roles, and obligations on HIV-related factors specifically for MSM that identified as Puerto Rican (PR MSM). This study aims to determine if family influences behaviors for young adult PR MSM related to condom use, HIV testing, health seeking behaviors, substance use, and sexual partners. A qualitative research approach is imperative for PR MSM to share their story to develop culturally informed interventions that are strengths-based for curtailing HIV incidence among Hispanic sexual minorities.

## Materials and methods

Academic Institutional Review Boards at Yale University and Texas State University approved the Dímelo (tell me about it) study that was administered throughout June and July 2015. Local community-based partnerships were established with the Puerto Rico Community Network for Clinical Services Research and Health Advancement (PR CoNCRA) and Comisión de Derechos Civiles de Puerto Rico. Community-based partners provided a private office to accommodate participants and administer face-to-face interviews in a private office setting.

An exploratory-descriptive qualitative (EDQ) design was used to gain insight on an understudied phenomenon [26]. This study explored five domains (I. Identity; II. Familism; III. Religiosity and Spirituality; IV. Community; and V. HIV Testing and Engagement to Care) on how cultural factors affect susceptibility to HIV transmission among young adult PR MSM ages 21–30 in San Juan, PR (refer to S1 File for the English and Spanish semi-structured interview). The descriptive component of EDQ took into account the literature to identify gaps of knowledge of a phenomenon for a particular population [26]. Development of the semi-structured interview was guided by the literature focused on family, *familismo*/familism, parental influence, and HIV-related factors for Hispanic MSM [21, 23, 27, 28]. A focus was placed on family as a unit as well as the interpersonal relationships of participants with immediate (e.g., parent, caregiver, sibling) and extended family members (e.g., aunt, uncle, cousin, grandparent). *Fictive kin*, unrelated by birth or marriage, was also explored to understand how participants applied family roles to important members within their social network [9]. Family dynamics play a pivotal role throughout an individual’s milestones and the age cohort 21–30 is important for this study to determine the implications of family and HIV-related factors for young adult PR MSM. The study was administered in San Juan, PR to circumvent issues related to receiving culturally informed HIV services by the community for the community, which raises the significance of this study’s partnership with PR CoNCRA and the Comisión de Derechos Civiles de Puerto Rico. The semi-structured interviews were originally written in English and the Comisión de Derechos Civiles de Puerto Rico provided a professional to translate the questions from English to Spanish. The Principal Investigator (PI; author MG) in addition to two other native Spanish speakers reviewed the Spanish versions to ensure accuracy.

Purposeful sampling was used to enhance representation of the priority population [26] by: 1) partnering with PR CoNCRA an established local HIV service provider; 2) hiring local bilingual PR MSM HIV service providers affiliated with PR CoNCRA who are actively involved in the MSM community; 3) advertising the study at gay social venues and gay social meeting applications (e.g., Grindr, Adam4Adam, Scruff); and 4) requiring people interested in the study to prescreen for eligibility. Participants eligible for the study were required to identify as Puerto Rican, cisgender male (participants specified their sex assigned at birth and their current gender identity), between the ages of 21 through 30, reside in San Juan, and report a sexual encounter with another male in the past six months. The community-based partners were in central locations throughout the city to increase accessibility and provide options for participants to complete the study. Partnership with the Comisión de Derechos Civiles de Puerto Rico was important to provide a safe space for participants concerned about HIV stigma and increase awareness on the organization’s role in protecting civil rights in Puerto Rico.

Prior to commencing the interview, the PI provided a written informed consent and verbally reviewed the consent form with the participant in person. The consent process informed participants that the purpose of the study was to explore how culture affects HIV-related factors among PR MSM in San Juan, PR. Participants were notified that the interview will be audio-recorded, last approximately 90 minutes, and receive a $50.00 cash incentive for completing the interview session. A unique number was assigned for each audio recording and signed consent forms were maintained in a separate locked file cabinet. The consent form also informed participants that results published for this study will not include any identifiable information. Titles based on relationship to the participant (e.g., mother, father, friend) were assigned for any names disclosed during the interview to protect the privacy of the participant and social network.

The PI administered all the semi-structured interviews to ensure the quality of information being collected and determine criteria for meeting saturation. Data saturation was determined when sufficient interviews were administered to obtain information related to the phenomena and no additional knowledge was gained from the sample population [29]. Guidelines for the number of participants to interview were based on the EDQ average sample size of 15 [26] and coding of the data to determine no new themes were identified for the phenomena [29].

**Fig 1:**
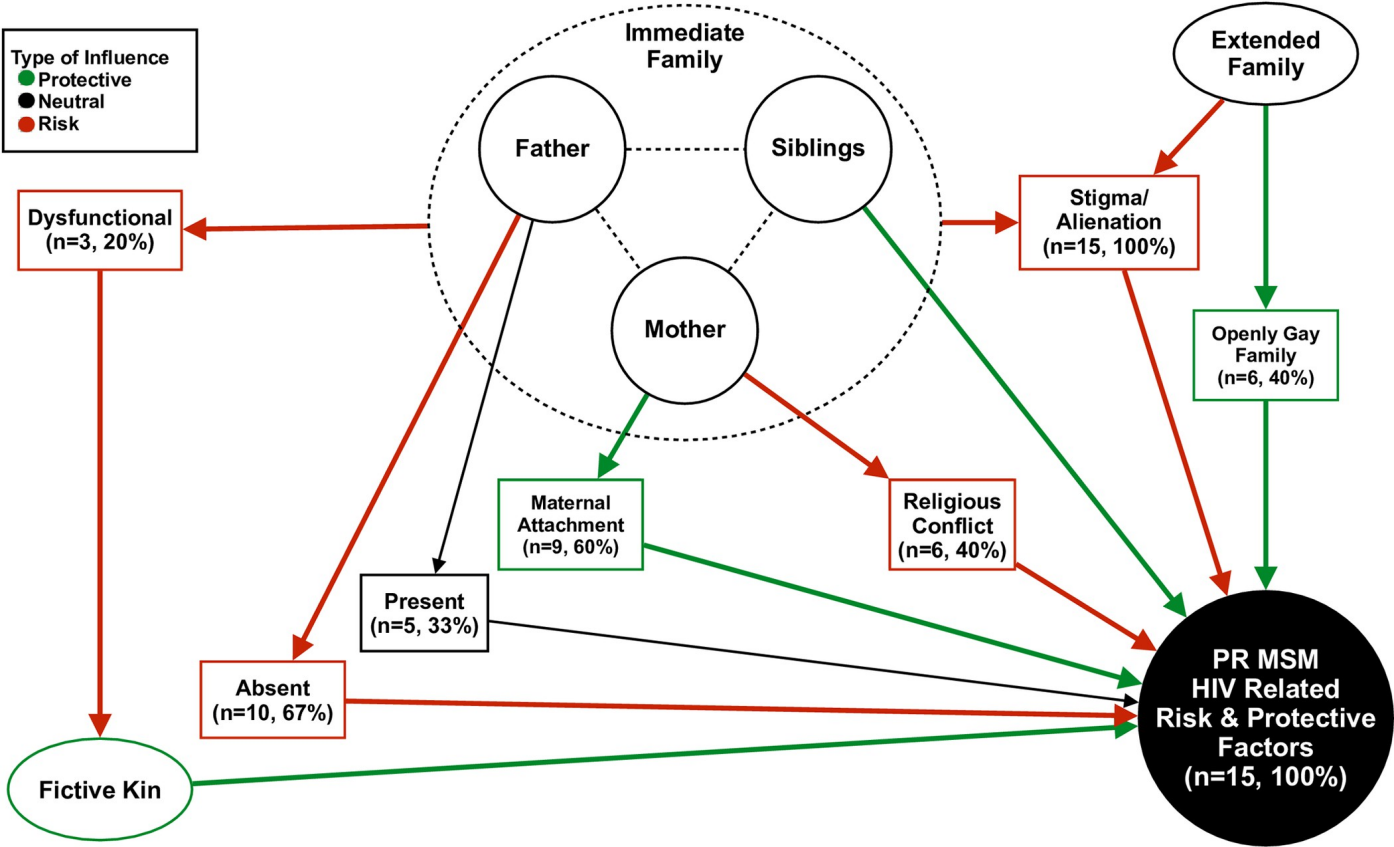
Thematic framework for conceptualizing family-based HIV related risk & protective factors for PR MSM (N = 15) in San Juan, PR.

A thematic analysis was administered solely by the PI based on Braun and Clarke’s [30] recommended phases for EDQ [26]: 1) Becoming familiar with the data; 2) Generating initial codes; 3) Searching for themes; 4) Reviewing themes; 5) Defining themes; and 6) Summarizing research findings. An inductive approach to thematic data analysis was applied to ensure that identification of the themes was data-driven and not influenced by preconceived theoretical implications related to the phenomenon of interest [30]. Prior to coding the data, it was important to reinforce a semantic approach based on the participants shared experiences related to the phenomenon [30] and codes initially were applied to any references made by the participant to biological family members and fictive kin. The PI’s intimate role of data collection, reading, cleaning, and coding of the data provided an opportunity to become deeply immersed and familiar with the participants lived experiences. NVivo software [31] was used to conduct a thematic analysis focused on identifying prominent themes based on codes associated with family. All data was transcribed verbatim, analyzed in its original language, and direct quotes were translated from Spanish to English by the PI. Generating initial codes involved a process of coding the first three interviews to identify codes related to the social phenomenon, which became a systemic process throughout the entire coding of the transcripts [30]. NVivo software [31] was used to extract data that was coded for family members and fictive kin to search for potential themes [30]. Potential themes were further reviewed to develop a thematic map to further define and conceptualize themes associated with the social phenomenon [30], refer to Fig 1. The final stage involved streamlining prominent themes to ensure rigor and credibility for conceptualizing implications of family members and fictive kin have on HIV related factors for PR MSM.

## Results

Table 1 provides demographic information of participants. A thematic map on family-based HIV risk and protective factors for PR MSM (Fig 1) was developed to provide a framework based on the following general themes: 1) Immediate versus Extended Family; 2) The Matriarch; 3) Fractured Paternal Relationships; 4) Siblings Influence; and 5) *Fictive Kin*–Creating My Own Family. Pseudonyms were used to protect the identity of participants in the following quotes.

**Table 1:** Participant demographics for Hispanic MSM (mean age = 26.2) in San Juan, PR.

Participant Demographics (N = 15)		
**HIV Status**		n (%)
	Negative	12 (80%)
	Positive	3 (20%)
**Ethnicity**		
	Puerto Rican	14 (93%)
	Puerto Rican-Black	1 (7%)
**Sexual Identity**		
	Gay	14 (93%)
	Bisexual	1 (7%)
**Household Income**		
	Under 9,999	3 (20%)
	10,000–19,999	7 (46%)
	20,000–29,999	3 (20%)
	30,000–39,999	1 (7%)
	40,000–49,999	1 (7%)
**Education**		
	HS Diploma	1 (7%)
	Professional Trade	1 (7%)
	Some College	5 (33%)
	Bachelor’s Degree	7 (46%)

### Immediate versus extended family

The majority of participants emphasized the important role immediate family members have on issues related to support and their high regard for family.

Angel: *With the extended family there is no support to a large extent…My nuclear family are my parents and my siblings*, *yes*. *But we* [extended family] *get along well*. *There is a mutual cordial relationship and we get along well*, *but it’s not at the level of “Wow*, *if you need something come and I will help you*!*” Not so much like that*, *it is not a relationship with greater assurance like there is with my close core* [immediate family].

Jose: *My* [immediate] *family always supports me*. *Always*, *almost always at 100% in whatever my personality brings forth*.

Juan: *It’s my* [immediate] *family*, *they’re blood* [laughs]. *Friends come and go*, *not family*. *In reality*, *my brothers and mother*, *for me I don’t care what people say*, *but what family is for me*, *my brothers*, *my mother*. *That’s it*.

All of the participants shared direct and indirect experiences of family members stigmatizing them for being gay.

Julio: *At the family level*, *mom may accept you*, *but there will always be someone in the* [extended] *family that sees you as*, *“That’s the son of* [participant’s mother], *the one that is gay*.*” Perhaps some uncle may look at you with reservations*. *So how will I take a person–my friends*, *my partner*, *my family–without fear that someone will say something or give a strange look or make an inappropriate comment*? *In other words*, *there are very few families that have a general openness towards a gay/lesbian topic*.

David: *And like your parents or the family would see it as*: *“Ah you are* [David], *the gay*.*” I am not* [David] *the gay*, *I am* [David]. *When you see your aunt*, *do you say*: *“My aunt*, *the heterosexual*.*” It’s important for me because it is who I am*, *it is the way I satisfy myself sexually and emotionally*, *but it’s like the whole world*. *I see it often*.

Santos: *First*, *she* [aunt] *would be like… He is a “pato”* [duck—derogatory slang for gay]! *And me*, *He’s not a “pato*,*” “pato” is an animal… Well*, *I was like*, *“that guy is a ‘pato’” and “you are a bitch*.*” “How are you going to call me a bitch if I’m your aunt*?*” I was like*, *I am not saying that you are or aren’t*. *You’re not that and they aren’t “patos*.*” I belong to that community*, *I am homosexual*, *I am gay*. *Those two words*, *I would love if you use them*, *but “pato” doesn’t sound very nice*.

All the participants expressed anxiety about coming out to their parent, regardless of having an openly gay extended family member that was accepted by the family. Some of the participants expressed how openly gay family members raised awareness on issues related to gender and sexuality, which was an important HIV protective factor for PR MSM.

Juan: *I didn’t think about saying it…and when I arrived home*, *she* [mother] *asked me if something happened to me*, *and I don’t know*, *it was like she smothered me and I told her yes*. *And she told me like what*, *“Tell me what is happening to you*. *Let’s go sit*.*” And until I told her*. *I told her that I was a homosexual*. *The first thing she told me was to protect myself*, *now that an uncle of mine* [that was openly gay] *has passed away from HIV/AIDS*. *That affected her a lot*. *She told me this*, *to take care of myself and not to dress like a woman*. *And that*, *she will always support me in everything*, *and from that day forward*, *since I told her that I am a homosexual*, *all of my family is very close with me*.

David: *Yes*, *“she’s” like a good friend* [referring to his gay uncle; laughs] *to me*. *And he calls me very often*. *With him*, *I have a relationship that if he calls me now*: *“What are you doing*, *are you going to the park*?*”*, *or something like that*. *It has turned into*, *now*, *at this moment*, *into something very close*. *But he was absent for about two years and he came around three weeks ago with this boom that “Look*, *I got AIDS*, *I’m scared*, *what should I do*?*” And now I’m returning to having this relationship with him–look he’s calling me right now–but with this emergency*, *I’m the only person he found and who he could confide in*, *it was with me*.

Tito: *I have helped in question to “he”* [referring to his cousin that has transitioned from male to female] *already knows that I know and “he” has told me about “his” sex change*. *I have sent clothes from here*, *women’s clothes*, *I have sent wigs*, *I have sent makeup*, *helped “him” in regards to accepting “himself” more*, *and if “he” wants to be like that*, *I will support “him” 100%*. *And if I need to help “him*,*” I’ll help “him” because “he” is my cousin*, *we are blood*. *And I know what “he’s” going through*, *not in regard to I’ve been through it*, *more like I know “his” father*, *I know in the way and pressure he has lived*.

### The Matriarch

Most of the participants emphasized how their mother played a critical role in providing a nurturing and supportive environment.

Victor: *My mother was always dedicated to us*, *she always gave us her best*, *she always worked for us*, *she always provided for us*. *Tremendous*, *excellent mother*.

Tito: *My mother always raised us with love and…we can count on her*. *It came out of me and I declared myself* [gay] *before her and her words were*: *“If I loved you before*, *I love you more now because you were honest with me*, *and I don’t care who you are*, *I will continue to love and support you in whatever*.*”*

Ricardo: *Totally*, *I actually told her* [mom] *during a family gathering*, *I said thank you for the childhood you have given me*. *Because I dealt with the good and I rejected the bad*. *My mother is very strict…and I am grateful that she has raised me that way because I know how to defend myself from the world and I know what I fall into…If I am a good person*, *if I am bad*, *what I’m involved with*. *I know that this is correct*, *that is bad*, *because my mother at one moment told me “Don’t do it*.*”*

Some of the openly gay participants expressed how their mothers would encourage them to practice safe sex.

Marcos: *My mother understands*, *she knows and she always tells me the three rules before I leave the house*: *key*, *wallet*, *and condoms* [laughs]. *She always does that to me*: *“What are the three*?*” and me*: *wallet*, *key*, *and condoms*.

Angel: *My mother*, *for example*, *is always like*: *“Remember to use a condom*, *and protect yourself*, *take the test”; but she always said it like “Oh look*, *at that*.*” But I do*, *clearly*, *I definitely listen*, *but it’s not like something that we talk about often among all of us*.

Santos was 16 years old when an older man sexually abused him. He saw the man one year later in public and told Santos “Welcome to the world of AIDS!” Santos refused to tell his mother about the sexual abuse, but shortly after the encounter he got tested and informed his mother that he is HIV positive.

Santos: *…I never mentioned the sexual abuse and it* [being HIV positive] *just changed our state of being and every night she* [mother] *would give me a massage*. *She would look after me all night; she hardly slept*. *I was also in the process*, *perhaps the depression in a way or sadness*. *I was also very isolated*. *But one day I saw her and she looked so sad…And I understood that as long as I am good*, *she is good as well*. *So nothing was the same*, *I went to receive treatment*, *I enrolled with a clinic*, *I started to look for information*, *and I started to become more optimistic*, *a lot more–thinking positive*.

Most PR MSM that described their mothers as religious were more likely to encounter homophobic attitudes and less likely to talk about their sexuality to avoid conflict with their mother.

Tony: *No*, *up to this day*, *my mother has no idea of my sexual preference and we don’t even talk about the subject*. *She is very Catholic and there are times when she makes homophobic comments*, *but she is my mother and I accept it for what it is*. *She was the person that gave me life*. *And that’s why*, *to avoid conflict*, *I don’t have that type of confidence to tell my mother of the way that I am or how I feel*.

Daniel: *She* [mother] *would say that she respected it*, *but it was a sin*, *it was unacceptable for people to have them sexual practices or whatever they would call it*. *And before I told her*, *I would feel a little bad that she would think that way*.

Julio: *…my mother kicked me out of the house for being a homosexual and for studying theatre*. *At the religious level*, *she is very conservative*, *she’s a Jehovah Witness; everything was very repressive*, *everything had its limits*. *One day she arrived at the house and she kicks me out*. *I was left on the streets*, *that best friend was the one that picked me up*, *the one I call “best friend” my brother*. *I am always bothered why these injustices occur*.

### Fractured paternal relationships

Some PR MSM reported that their father was present, but only a few participants described supportive relationships with their father as a young adult. Participants clarified that their fathers provided tangible support, but they were not emotionally available.

Angel: *…In regard to my father*, [we talk] *sometimes over work*. *About situations that occur at work*, *like “Look*, *this happened to me*, *what do you think*?*” Also*, *we don’t talk much*, *but we talk every now and then*.

Julio: *The image of my father was purely financial*. *When I had a problem*, *he would be able to provide financially because he was always working*. *He is a very formal man… he demonstrated affection through toys*, *giving me food*, *and taking me out to eat*.

Miguel: *She* [mother] *was caring and nurturing*, *more patient*. *He* [father] *was more rough*, *demanding*. *I would say throughout my life*, *she* [mother] *was the one who was more keen on what I was interested in doing and seeing how she could either be involved or help me achieve doing stuff with that interest*. *Whereas*, *usually*, *he wanted to do something and it had to be his way or I had to do what he wanted me to do*. *So they were opposites in that sense…my dad would be the one who got pissed and yelled and cussed when you didn’t do well in school*. *My mom was the one who would take me to school*, *who would want to talk to the teachers*, *and who was overtly acknowledging when I did well*.

The majority of PR MSM reported that their fathers were absent and did not play a critical role in their upbringing. However, most PR MSM shared memories of their father being homophobic and experiencing risk factors such as a history of child abuse and being kicked out of the house for being gay.

David: *Because my father came to the house* [and kicked me out], *I never lived with him*, *and I never had a relationship with him*, *he was very mean*.

Luis: *…my father was a highly homophobic person; I think he always knew that I was gay because he would always come by to tell me that he would never accept a gay son and he was always stern… I was afraid of his reaction*, *a strong fear of what his reaction would have been*, *so I escaped from my house and never returned… He beat me a lot as a kid*, *that’s why I escaped from my house because I was scared of his reaction*, *he could have killed me; because they weren’t like any other beatings*, *they were extremely harsh*.

Marcos: *My father*, *no*. *He was in my life in different stages*, *but he was a person that was never stable*. *He was a person that would go*, *come*, *enter*, *leave*, *he would get lost*. *My father had problems with alcohol*.

### Siblings influence

The majority of PR MSM reported that their siblings supported them and were concerned for their well-being.

Angel: *I have three siblings older than me*. *I think because I’m the youngest in the family*, *they were always on top of me*: *“You have to do this*, *you have to do that*, *follow this example*.*” Because they always wanted me to be a good person in society and in reality; they all influenced me a lot*.

Juan: *They* [siblings] *are always worried about me*, *like any other older brother*, *whatever he says*. *He is always supporting me*.

Luis: *…I had my mannerisms and effeminate behaviors ever since I was a kid; it was always consistent*. *And well yes*, *I had a lot of bullying in school as a kid…They* [brothers] *had to defend me and all because I was more delicate*, *more feminine*, *and they* [people at school] *always bullied me*.

Jose: *…they* [siblings] *always showed me not to use drugs*. *That I would encounter in life people that will always offer me drugs*, *but to try not to do it*. *They always explained the consequences of that*.

However, one participant indicated that observing siblings using drugs and having multiple sex partners increased the likeliness of him doing the same.

Marcos: *Yes*, *for me it was normal*. *I remember that my brother would throw parties*, *he would invite half the school when mom would leave for the weekend; and the house would be full of people up to the rooftop drinking alcohol… For example*, *just like them* [brothers] *you could say that I am “super promiscuous*.*” Me*, *if I’m bored at home and I have a cell phone and I log into Grindr*, *I could have sex with four different persons on the same day*.

### Fictive Kin–creating my own family

The immediate family played an integral role, but only a few participants described their family as dysfunctional and being disowned due to their sexuality. However, PR MSM demonstrated their resilience by creating their own family through *fictive kin* to establish a meaningful supportive network.

Luis: *I have been able to create family with my friends*. *My best friend for me… he is family*. *He’s someone I support*, *he supports me*. *And if I need anything or if he would need anything*, *be it financially*, *emotionally… something can happen to me*, *for example an accident*. *Not to be so tragic*, *but when they ask me for a telephone number just in case something happens at work or the university*, *the number I give them is my best friend*. *I consider that is the family that I have been able to develop in my life*.

Two out of the three participants seroconverted when they were homeless during their adolescence. Julio, a PLWH, shares the following story when he was kicked out of his biological mother’s home for being gay and refers to his stepmother (who was separated from his father) as his mother.

Julio: *She* [stepmother] *up to this day has said*: *“It hurt me a lot*. *It was like they ripped something out of me*. *But how was I going to be the falling tree*, *firewood*?*” I then came down*, *we ate; the subject was not discussed*, *like for two weeks*. *She came with me to CoNCRA* [HIV/AIDS organization] *for the first interview and she always provided moral support*. *“You are not alone; you are with me*. *Your grandmother loves you*. *Your brother loves you*.*” The razor blades–she would eat with me–she wouldn’t change the razor blades in the bathroom*. *“Everything will be the same*. *This is your toothbrush*, *this is* [refers to stepbrother].*” Normal*. *But she didn’t change the cup*, *the plates*, *everything normal*. *It’s normal*. *And I was assimilated little by little with my condition with being normal*.

## Discussion

The purpose of this study was to examine the role family members have on HIV-related risk and protective factors for PR MSM in San Juan, PR. Prior research has focused on the implications of family among adolescent sexual minorities [7, 23, 27, 28, 32–34], but this study provides a novel approach by focusing on young adults (ages 21–30) towards an in depth understanding of cultural beliefs on the meaning of family for PR MSM. Research findings for this study found that the immediate family was distinguished from the extended family as a reliable source of support for enhancing HIV-related protective factors for young adult PR MSM. The thematic map (Fig 1) provides an innovative framework to conceptualize the influence interpersonal family relationships have for the well-being of PR MSM. This study makes a significant contribution to research by further conceptualizing the meaning of family and identifying the significant role mothers, siblings, *fictive kin*, and openly gay family members have in enhancing HIV-related protective factors (e.g., condom use, HIV testing) for young adult PR MSM.

*Familismo/*familism has been identified as a prominent cultural factor for Hispanics, which reinforces a collectivistic culture for family members to support one another and prioritize the needs of the family [12, 35]. However, the effects of *familismo/*familism have been attributed to the overall family (i.e., immediate family, extended family, *fictive kin*) and conceptualized for the general Hispanic population, which limits important cultural nuances associated with diverse national Hispanic identities such as Puerto Ricans [35]. PR MSM in this study prioritized maintaining their relationships with their immediate family and as a protective factor they refrained from sharing personal information with extended family members to avoid conflict. This study reinforces prior research findings that *familismo*/familism is composed of complex social networks that become more challenging for Hispanic sexual minorities to prioritize family needs over personal needs, especially related to one’s sexuality for sexual minority family members [36]. As demonstrated by Muñoz-Laboy [36], comparison of the findings revealed similar results, PR MSM in this study indicated that their sexuality threatened their family foundation of support and interconnectedness. PR MSM in this study expressed more concern about their immediate family and less concern about extended family members.

Fear of family rejection for Hispanic MSM causes conflict for individuals because they want to maintain family ideals and avoid parent disapproval [6, 37]. Studies have demonstrated that family support enhances protective factors and family marginalization increases risk factors for sexual minorities [12, 38]. For example, PR MSM for this study that reported being accepted by their mother during the coming out process were encouraged by their mother to use condoms and get tested for HIV. Researchers have indicated that mothers who address same-sex behavior is an important factor for reducing at-risk behavior for sexual minorities [23]. However, a few participants reported being disowned from their family when their parents discovered that their child was a sexual minority. PR MSM in this study revealed that *fictive kin* became a critical source of support when they were shunned from their biological family. The literature on *familismo*/familism indicates that Hispanics have extensive networks that commonly include *fictive kin* as a protective factor [9]. However, PR MSM in this study were considerate when they referenced family members and only a few participants that were disowned from their family identified *fictive kin* as an important source of support. As one participant indicated in this study, his nonbiological mother supported him in accessing HIV services when he seroconverted. This study raises the significance of family for young adult PR MSM and the importance for creating their chosen family through *fictive kin* as a protective factor to enhance their well-being.

A greater emphasis needs to be placed on the importance of family structure and implications for the well-being of PR MSM being raised by one or both parents. PR MSM that reported being raised by both parents emphasized that their father played a critical role as a financial provider, which enhanced access to resources. However, despite PR MSM having a father present in their lives, they solely relied on their mother for emotional support. Findings for this study corroborate other research findings that sexual minorities report stronger relationships with their mother compared to their father [39]. Findings for this study also supported other research findings that their father-son relationship deteriorates when their father suspects them of being gay [34], which usually occurred during adolescence for PR MSM in this study. Another important distinction was that fathers that did not reside in the same household were more likely to be described as authoritarian and mothers would rely on them to discipline their children. Findings suggest that it may be more important to focus on the influence parents have on the well-being of Hispanic sexual minorities as opposed to the overall family.

An unanticipated finding was that the majority of PR MSM reported being raised in a female-headed household and only one-third indicated that their father was present in their lives. Female-headed households are more likely to be poverty-stricken [40], which is an important risk factor to consider in the development of sexual minority children. An important finding for this study revealed that maternal support, regardless of having a father present, was an influential factor for young adult PR MSM in determining risk and protective factors associated with *familismo*/familism. Perceived immediate family cohesion and maternal attachment were critical HIV protective factors for PR MSM. This may explain prior research findings indicating mixed outcomes associated with *familismo*/familism [9, 12]. This study suggests family-based interventions to place greater attention on perceived maternal support to mitigate susceptibility to HIV among diverse Hispanic sexual minorities.

Another important finding was that religious beliefs hindered the mother-son relationship during the coming out process. PR MSM that referred to their mothers as highly religious were more likely to experience externalized and internalized homophobia. Findings for this study corroborate other research findings of Hispanic sexual minority family members prioritizing the needs of the family and avoiding any reference of their sexuality to maintain existing familial relationships [12, 36, 38]. The internalized conflict PR MSM experienced in this study raises important concerns associated with the influence homophobic religious rhetoric has on one’s well-being [12, 41]. As one research participant expressed, it was a sin to be gay, which made him feel bad about his sexuality. However, PR MSM expressed that extended family members who openly identified as a sexual minority played an important role in supporting one another and creating a caring family environment for sexual minorities. PR MSM in this study shared experiences on how they would confront extended family members when they were stigmatized for being gay. For example, a participant described an encounter with his aunt using “*pato*” as a derogatory reference in Spanish of sexual minorities and he emphasized to his aunt the significance of using respectful terms based on one’s sexuality as gay or homosexual. Findings for this study suggest that sexual minority family members that are out play a critical role in addressing direct and indirect stigmatization of sexual minorities.

This study’s findings also identified straight siblings as an important protective factor for sexual minority family members. This study corroborated existing research on the protective role straight siblings have in providing support, guidance, and protection of sexual minority siblings [42]. Straight siblings would reinforce a supportive environment for PR MSM by coming to their defense if they encountered bullying for being a sexual minority and encouraged them not to use drugs, which are important factors associated with HIV susceptibility. A recent study also indicated that Black youth who perceived a loving relationship with their sibling were more likely to get tested for HIV [39]. However, as a participant indicated for this study that siblings may also have a negative influence regarding their substance use and sexual behaviors, which may increase susceptibility to HIV. Research findings suggest that family-based HIV interventions should support the role of straight siblings as allies to enhance HIV-related protected factors for sexual minority siblings.

Study limitations are due to purposeful sampling, sample size, and residential area. The analysis was conducted solely by the PI and efforts as discussed in the methods section were made to reinforce a data-driven approach to identify prominent themes. Also, stratified sampling for HIV status was not taken into consideration, which resulted in three PLWH in this study. PLWH encounter a different experience that is worth exploring to further understand the influence interpersonal encounters with family members has on the well-being of Hispanic MSM. Despite limitations, the methodology for qualitative thematic analysis provides an important research tool for conveying critical insight on a social phenomenon [30] towards understanding how family affects the lives of Hispanic MSM.

## Conclusions

The research findings for this study provide an understanding of family and the important role individual family members have on reinforcing HIV-related risk and protective factors for PR MSM between the ages of 21–30. Further research is required on exploring the parental influence for young adult Hispanic MSM from diverse nationalities (e.g., Mexican, Cuban), but based on the findings it is recommended that HIV services develop opportunities for family-based interventions that enhance maternal relationships. The immediate family in particular requires support towards empowering Hispanic MSM to practice healthy behaviors regarding substance use, condom use, HIV testing, access and engagement in health care. Finally, the immediate family for Hispanic MSM requires assistance to mitigate sexual minority family members from feeling stigmatized and isolated. Overall, family-based interventions reinforce cultural beliefs and values through a strengths-based approach towards enhancing supportive relationships for Hispanic MSM and reducing HIV risk behaviors.

## Supporting information

S1 File (DOCX)
