# Supplementary material for: “You are not alone”: Family-based HIV risk and protective factors for Hispanic/Latino men who have sex with men in San Juan, PR
Source: PLoS One. 2022 Jun 16;17(6):e0268742. doi: 10.1371/journal.pone.0268742 (PMC9202870; doi:10.1371/journal.pone.0268742)
Supplement: S1 File — (DOCX) [file pone.0268742.s001.docx]

**Supplement 1** Dímelo (tell me about it) Research Interview Guide in English and Spanish (*items in italics*)

**I. Identity/*Identidad***

1. Let’s get started by you describing who you are or how you would describe or present yourself to others?

*¿Vamos a comenzar pidiéndole que describa quién es usted o cómo se describiría o presentaría ante otras personas?*

1. What are some important personal traits that define who you are and how are they reinforced?
   1. Does your race or ethnicity play a role, how?
   2. Do your religious or spiritual beliefs and values influence who you are, how?

*¿Cuáles son algunas características personales importantes que definen quién es usted y cómo se refuerzan?*

1. *¿Desempeña algún papel su raza o etnia? ¿Cómo?*
2. *Sus creencias y valores religiosos o espirituales, ¿influyen en quién es usted? ¿Cómo?*
3. How would you describe your ideal self or how would you like other people to view you?

*¿Cómo describiría su imagen ideal de usted mismo o cómo le gustaría que otras personas lo percibieran?*

1. What influence has your family (specify who) played in helping you become who you are and how?

*¿Qué influencia ha tenido su familia (especifique quién en particular) en ayudarlo a convertirse en la persona que es hoy y cómo lo ha influenciado?*

1. How does your sexuality influence who you are and your identity?

*¿Qué influencia tiene su sexualidad en quién es usted y su identidad?*

**II. Familism*/Familismo***

1. Why don’t we get started by you describing your immediate family (specify, members in the family, influence they have on your life) – remember not to use names just their role (e.g., mom, dad, brother)?

*Vamos a comenzar describiendo a su familia inmediata (especifique los miembros de su familia, la influencia que tienen en su vida) – recuerde no utilizar nombres, sólo el papel que desempeñan (por ej., mamá, papá, hermano).*

1. What does *respeto* mean to you?

*¿Qué quiere decir respeto para usted?*

1. How does the family influence the meaning of *respeto*?
   1. Would you say that *respeto* influences how you behave in public or what people would think of you?
   2. Explain how or why not?

*¿Qué influencia tiene su familia en el significado de respeto?*

1. *¿Cree usted que el respeto influye en cómo usted se comporta en público o qué piensa la gente de usted?*
2. *Explique cómo o por qué no.*
3. What role does your sexuality have on how your family perceives you?
   1. Do you change your behavior or how you act to gain their approval?
   2. What influence does this have on you?

*¿Qué papel desempeña su sexualidad en la manera en la que lo percibe su familia?*

1. *¿Cambia usted su comportamiento o la forma en la que actúa para obtener la aprobación de su familia?*
2. *¿Qué influencia tiene esto en usted?*
3. Explain how your family played a role in using or not using drugs…
   1. drinking alcohol
   2. smoking tobacco
   3. smoking marijuana
   4. injecting drugs (specify drug, e.g. heroin)

*Explique cómo su familia desempeñó un papel en su uso o no uso de drogas…*

1. *consumir alcohol*
2. *fumar tabaco*
3. *fumar marihuana*
4. *inyectarse drogas (especifique qué droga, por ej. heroína)*
5. How comfortable would you feel to go to your family for advice and why?
   1. Who would be the best person in your family to talk about issues related to your sexuality or sex? Explain why…
   2. Who would be the best person in your family to talk about drug use? Explain why…
   3. Would you feel comfortable telling your family that you are gay or bisexual?
   4. Would you feel comfortable telling your family if you got infected with HIV?

*¿Cuán cómodo se sentiría usted pidiéndole consejos a su familia y por qué?*

1. *¿Quién sería la mejor persona en su familia para hablar de asuntos relacionados con su sexualidad o el sexo? Explique por qué…*
2. *¿Quién sería la mejor persona en su familia para hablar de asuntos relacionados con el uso de drogas? Explique por qué…*
3. *¿Se sentiría cómodo diciéndole a su familia que usted es homosexual o bisexual?*
4. *Si usted estuviera infectado con el VIH, ¿se sentiría cómodo diciéndoselo a su familia?*
5. What role does your family play in encouraging you to stay healthy and how?

*¿Qué papel desempeña su familia en animarlo a mantenerse saludable y cómo lo hace?*

**III. Religiosity and Spirituality**/***Religiosidad y espiritualidad***

1. How would you describe in your own words the meaning of being religious and/or being spiritual?
   1. How are they the same?
   2. How are they different?

*¿Cómo definiría en sus propias palabras lo que es ser religioso y/o espiritual?*

1. *¿En qué se parecen?*
2. *¿Cuál es la diferencia?*
3. Currently, what influence does **religion** have on your life?
4. How does your family reinforce your religious beliefs or values?
5. Which family member would you say plays a significant role in your religious beliefs?
6. Has there been a difference in your religious beliefs as a child compared to now?

*Actualmente, ¿qué influencia tiene la* ***religión*** *en su vida?*

1. *¿Cómo refuerza su familia sus creencias o valores religiosos?*
2. *¿Qué miembro de su familia diría usted que desempeña un papel significativo en sus creencias religiosas?*
3. *¿Han cambiado de alguna forma sus creencias religiosas desde que usted era un niño hasta ahora?*
4. Currently, what influence does **spirituality** have on your life?
5. How does your family reinforce spiritual beliefs?
6. Which family member would you say played a significant role in your spiritual beliefs?
7. Has there been a difference in your spiritual beliefs as a child compared to now?

*Actualmente, ¿qué influencia tiene la* ***espiritualidad*** *en su vida?*

1. *¿Cómo refuerza su familia sus creencias espirituales?*
2. *¿Qué miembro de su familia diría usted que desempeña un papel significativo en sus creencias espirituales?*
3. *¿Han cambiado de alguna forma sus creencias espirituales desde que usted era un niño hasta ahora?*
4. How has religion or spirituality influenced your overall wellbeing and/or health?

*¿Cómo ha influenciado la religión o la espiritualidad en su bienestar general o salud?*

1. How has religion or spirituality influenced your thoughts about your sexuality?

- What about sex (sexual behaviors, activity, and or encounters)?

*¿Cómo ha influenciado la religión o la espiritualidad sus opiniones sobre su sexualidad?*

- *¿Y en cuanto al sexo (comportamientos, actividad y/o relaciones sexuales)?*

1. How has religion or spirituality influenced your thoughts about using drugs?

*¿Cómo ha influenciado la religión o la espiritualidad sus opiniones en cuanto al uso de drogas?*

1. How comfortable would you feel to go to your church or your spiritual community for advice and why?
2. Does it depend on what type of advice (e.g., HIV, drug use, depression)?
3. How would your religious and/or spiritual community react if you told them you were gay or bisexual?
4. How would your religious and/or spiritual community react if you told them you were HIV positive?

*¿Cómo se sentiría usted pidiéndole consejos a su iglesia o comunidad espiritual y por qué?*

1. *¿Dependería del tipo de consejo (por ej. VIH, uso de drogas, depresión)?*
2. *¿Cómo reaccionaría su comunidad religiosa y/o espiritual si usted le dijera que es homosexual o bisexual?*
3. *¿Cómo reaccionaría su comunidad religiosa y/o espiritual si usted le dijera que es VIH positivo?*

**IV. Sense of Community/Sentido de Comunidad**

1. How would you describe your community overall?

*¿Cómo describiría su comunidad en términos generales?*

1. A community usually involves different groups of people that you might get along with or not get along with…let’s focus on the groups you get along with first. What groups of people in your life play an important role in supporting you?
   1. How do they affect your overall wellbeing?
   2. How do they influence your religious beliefs and values?
   3. How would they react if you told them that you were gay or bisexual?
   4. How would they react if you told them that you are HIV positive?

*Una comunidad por lo general comprende distintos grupos de personas con los que usted puede llevarse bien o mal… vamos a enfocarnos en los grupos con los que usted se lleva bien primero. ¿Qué grupos de personas en su vida desempeñan un papel significativo en brindarle apoyo?*

1. *¿Cómo afectan su bienestar general?*
2. *¿Cómo influyen en sus creencias y valores religiosos?*
3. *¿Cómo reaccionarían si usted les dijera que es homosexual o bisexual?*
4. *¿Cómo reaccionarían si usted les dijera que es VIH positivo?*
5. Now let’s focus on the groups in your life that may cause tension or negatively affect you?
   1. How do they affect your overall wellbeing?
   2. How do they influence your religious beliefs and values?
   3. How would they react if you told them that you were gay or bisexual?
   4. How would they react if you told them that you are HIV positive?

*Ahora, vamos a enfocarnos en los grupos en su vida que pueden ocasionar tensión o afectarlo de manera negativa.*

1. *¿Cómo afectan su bienestar general?*
2. *¿Cómo influyen en sus creencias y valores religiosos?*
3. *¿Cómo reaccionarían si usted les dijera que es homosexual o bisexual?*
4. *¿Cómo reaccionarían si usted les dijera que es VIH positivo?*

**V. HIV Testing and Engagement to Care/**

**Pruebas de VIH y compromiso con el cuidado**

1. What type of positive related HIV or social services (e.g., testing, treatment) have you received in San Juan?
   1. What in particular made those services a **GOOD** experience?
   2. What role did the staff play on addressing your needs?
   3. Was the cost for services a factor on the type of services you received?

*¿Qué tipo de servicios relacionados con el VIH o servicios sociales (por ej., pruebas, tratamiento) positivos ha recibido en San Juan?*

1. *¿Qué en particular hizo que esos servicios fueran una* ***BUENA*** *experiencia?*
2. *¿Qué papel desempeñaron los trabajadores en atender sus necesidades?*
3. *El costo de los servicios, ¿fue un factor que influyó en el tipo de servicios que usted recibió?*
4. What type of negative related HIV or social services have you received in San Juan?
   1. What in particular made those services a **BAD** experience?
   2. What role did the staff play on addressing your needs?
   3. Was the cost for services a factor on the type of services you received?

*¿Qué tipo de servicios relacionados con el VIH o servicios sociales negativos ha recibido en San Juan?*

1. *¿Qué en particular hizo que esos servicios fueran una* ***MALA*** *experiencia?*
2. *¿Qué papel desempeñaron los trabajadores en atender sus necesidades?*
3. *El costo de los servicios, ¿fue un factor que influyó en el tipo de servicios que usted recibió?*
4. What type of social services are needed in San Juan for men who have sex with men?

*¿Qué tipo de servicios sociales se necesitan en San Juan para los hombres que tienen sexo con hombres?*

1. What type of HIV related services specifically are needed in San Juan for men who have sex with men?

*¿Qué tipo de servicios relacionados con el VIH específicamente se necesitan en San Juan para los hombres que tienen sexo con hombres?*

1. Overall, do you have any suggestions on how to encourage men to get tested or maintain treatment for HIV in San Juan?

*En términos generales, ¿tiene usted alguna sugerencia sobre cómo animar a que los hombres se hagan la prueba del VIH o continúen recibiendo tratamiento para el VIH en San Juan?*

**Wrap-up**

1. Overall, based on what you have shared with me, what’s one thing that would be important for the research study to take into consideration?

*Generalmente hablando, según lo que usted ha compartido conmigo, ¿qué cosas debería tomar en consideración la investigación?*

1. Would you like us to consider any additional topic or area that you feel we may have overlooked?

*¿Le gustaría que consideráramos cualquier otro tema o área que crea que podamos haber omitido?*

1. Do you have any questions for me before we end the interview?

*¿Tiene alguna pregunta para mí antes de terminar la entrevista?*
